# Provider Attitudes toward the Voluntary Medical Male Circumcision Scale-Up in Kenya, South Africa, Tanzania and Zimbabwe

**DOI:** 10.1371/journal.pone.0082911

**Published:** 2014-05-06

**Authors:** Webster Mavhu, Sasha Frade, Ann-Marie Yongho, Margaret Farrell, Karin Hatzold, Michael Machaku, Mathews Onyango, Owen Mugurungi, Bennett Fimbo, Peter Cherutich, Dino Rech, Delivette Castor, Emmanuel Njeuhmeli, Jane T. Bertrand

**Affiliations:** 1 Zimbabwe AIDS Prevention Project-UZ, Department of Community Medicine UZ, Harare, Zimbabwe; 2 Centre for HIV and AIDS Prevention Studies, Johannesburg, South Africa; 3 Tulane School of Public Health and Tropical Medicine, Department of Global Health Systems and Development, New Orleans, LA, USA; 4 Population Services International, Harare, Zimbabwe; 5 Jhpiego, Dar es Salaam, Republic of Tanzania; 6 FHI 360, Kisumu, Kenya; 7 Ministry of Health and Child Welfare, Harare, Zimbabwe; 8 Ministry of Health and Social Welfare, Dar es Salaam, Republic of Tanzania; 9 National AIDS/STD Control Programme, Nairobi, Kenya; 10 United States Agency for International Development, Washington, DC, USA; World Health Organization, Switzerland

## Abstract

**Background:**

Countries participating in voluntary medical male circumcision (VMMC) scale-up have adopted most of six elements of surgical efficiency, depending on national policy. However, effective implementation of these elements largely depends on providers' attitudes and subsequent compliance. We explored the concordance between recommended practices and providers' perceptions toward the VMMC efficiency elements, in part to inform review of national policies.

**Methods and Findings:**

As part of Systematic Monitoring of the VMMC Scale-up (SYMMACS), we conducted a survey of VMMC providers in Kenya, South Africa, Tanzania, and Zimbabwe. SYMMACS assessed providers' attitudes and perceptions toward these elements in 2011 and 2012. A restricted analysis using 2012 data to calculate unadjusted odds ratios and 95% confidence intervals for the country effect on each attitudinal outcome was done using logistic regression. As only two countries allow more than one cadre to perform the surgical procedure, odds ratios looking at country effect were adjusted for cadre effect for these two countries. Qualitative data from open-ended responses were used to triangulate with quantitative analyses. This analysis showed concordance between each country's policies and provider attitudes toward the efficiency elements. One exception was task-shifting, which is not authorized in South Africa or Zimbabwe; providers across all countries approved this practice.

**Conclusions:**

The decision to adopt efficiency elements is often based on national policies. The concordance between the policies of each country and provider attitudes bodes well for compliance and effective implementation. However, study findings suggest that there may be need to consult providers when developing national policies.

## Introduction

Fourteen Eastern and Southern African countries are scaling-up voluntary medical male circumcision (VMMC) as part of a comprehensive HIV prevention strategy [1], [2], [3]. The World Health Organization (WHO) convened a panel of experts in 2009 and subsequently issued guidance on ways to improve efficiency while ensuring safety under the document titled, “Models for Optimizing Volume and Efficiency for Male Circumcision Services “or MC MOVE [4]. Whereas the document offers non-prescriptive “considerations” for how to improve efficiency and contextualize services, practitioners working with the scale-up subsequently identified six elements of surgical efficiency in VMMC: task-shifting, task-sharing, use of pre-bundled kits with disposable instruments, rotation between multiple beds, use of forceps-guided surgical method, and use of electrocautery. Countries participating in the scale-up have adopted most of these different elements [5].

Although studies have explored the impact of provider attitudes on the implementation of national policies in a number of health interventions (e.g., hypertension care [6], mental health management [7], malaria treatment [8], [9], [10] as well as provider-initiated HIV testing and counseling [11]), little research has been published to date on provider attitudes vis-à-vis the implementation of the VMMC scale-up. To our knowledge, there is only one publication that examines practices and attitudes of VMMC providers [12].

The objective of this analysis is to assess the attitudes of VMMC providers in four countries (Kenya, South Africa, Tanzania and Zimbabwe) toward these six elements of surgical efficiency. South Africa is the only country whose documented VMMC guidelines are up-to-date (see Table 1).

**Table 1:** VMMC strategies and plans in the four countries, and efficiency elements included in each country's national guidelines.

Country	Strategy	Year	Efficiency element included in national policy
Kenya	National Guidance for Voluntary Male Circumcision in Kenya	2007	Task-shifting – both doctors and nurses allowed to be primary providers
	National Strategy for Voluntary Medical Male Circumcision	2009	
South Africa	South African National Guidelines for Medical Male Circumcision Under Local Anesthesia	2011	Task-shifting – only doctors allowed to be primary providers but nurses allowed to perform certain tasks
			MC-MOVE the nationally-recommended practice (including all 6 efficiency elements – see comment above regarding task-shifting)
Tanzania	National Strategy for Scaling-up Male Circumcision for HIV Prevention	2010	Task-shifting – both doctors and nurses allowed to be primary providers
Zimbabwe	Zimbabwe Policy Guidelines on Safe and Voluntary Male Circumcision	2009	Task-shifting – only doctors allowed to be primary providers but policy recognizes the need to allow and train non-doctors to conduct VMMC
	Strategy for Safe Medical Male Circumcision Scale-Up to Support Comprehensive HIV Prevention in Zimbabwe	2010	Forceps-guided method the nationally-recommended surgical technique

Note: Kenya, South Africa and Zimbabwe's guidelines have not been updated to include other nationally-recommended VMMC practices.

## Methods

Human subjects approval was obtained by the Tulane University Institutional Review Board (IRB) and the local IRBs in each country: the Kenya Medical Research Institute, the University of the Witwatersrand's Human Research Ethics Committee (SA), the Tanzanian National Institute for Medical Research, and the Medical Research Council of Zimbabwe. Additional scientific and ethical reviews were done by the national AIDS coordinating bodies, other government officials, local NGOs, and other stakeholders

This is an analysis of data collected as part of the SYMMACS study which has been described elsewhere [5]. In brief, SYMMACS tracked the implementation and evolution of VMMC services in four countries. Data on the efficiency elements were collected at VMMC sites over a two-day period in 2011 (n = 73) and in 2012 (n = 122) across the four countries. A detailed description of how sites were selected is provided in the overview article [5] in this supplement. In brief, the four countries differed in the stage of their VMMC scale-up; this determined how sites were selected. Kenya, with over 235 sites operational by December 2010 randomly selected 30 sites for 2011 data collection. In 2012, four of these sites were replaced and one was dropped, resulting in 29 sites in 2012. By contrast, South Africa, Tanzania, and Zimbabwe had only 1–3 VMMC sites by late 2010. Each country identified and selected all known or planned sites for 2011. Some of these sites were replaced and more were added in 2012, depending on each country's circumstances [5].

In each country, the research team interviewed all providers involved in the clinical delivery of VMMC services on the two-day visit to each site in 2011 and 2012. The SYMMACS survey included a structured questionnaire which assessed provider attitudes, beliefs, and practices related to the six efficiency elements. At the end of each structured interview, the interviewer asked eight open-ended questions. These allowed for more in-depth responses on providers' experience with VMMC, in addition to providing the interviewer with an opportunity to seek clarification on specific responses.

Data were managed and analyzed using SPSS version 19.0. Responses to attitude questions were recoded from a five-point Likert scale ranging from “strongly disagree” to “strongly agree” to a binary outcome of “agree/strongly agree” vs. others. This was done to provide higher clarity between categories and to compensate for the potentially limiting sample size. Frequency counts and percentages were tabulated for categorical variables in the descriptive analysis. A chi-square test for independence or Fisher exact test when the expected cell frequency was lower than five was performed to determine if either cadre or country (the key correlates of interest) was associated with agreement/disagreement to each attitudinal question. These tests were performed and reported for each year separately.

A restricted analysis using 2012 data to calculate unadjusted odds ratios and 95% confidence intervals for the country effect on each attitudinal outcome was done using logistic regression. These analyses were restricted to data collected in 2012 due to the overlap in both sites and providers between the two years of data collection, and the inability (due to the manner of data collection) to distinguish unique providers. Additionally, unadjusted odds ratios were calculated. Qualitative data from open-ended responses were used to triangulate with the quantitative analyses.

## Results

In the four countries, a total of 358 and 591 providers took part in the SYMMACS study in 2011 and 2012, respectively. The socio-demographic profile of the providers is shown in Table 2, and described in detail by Perry et al. [13] in this supplement. Overall, in South Africa and Tanzania the majority of VMMC providers were female, whereas in Kenya and Zimbabwe females were the minority. In all countries where medical doctors provided VMMC, the cadre was predominately male.

**Table 2:** VMMC provider profile in the four SYMMACS countries.

	Kenya		South Africa		Tanzania		Zimbabwe	
	2011	2012	2011	2012	2011	2012	2011	2012
	n = 85	n = 82	n = 105	n = 209	n = 93	n = 206	n = 74	n = 94
**Gender:**								
Male (%)	80.0	69.5	45.7	41.6	32.3	39.8	67.6	67.0
**Mean age in years,**	32.0	31.0	38.5	39.1	40.2	40.4	39.3	37.7
(Standard deviation)	(6.7)	(6.6)	(9.8)	(10.9)	(9.4)	(8.8)	(8.4)	(8.7)
**Cadre:**								
Medical Doctor	0.0	0.0	20.0	16.7	0.0	1.9	25.7	26.6
Nurse	52.9	53.7	80.0	83.3	80.6	78.6	74.3	73.4
AMO	0.0	0.0	0.0	0.0	8.6	5.3	0.0	0.0
Clinical officer	47.1	45.1	0.0	0.0	10.8	14.1	0.0	0.0
**Role in surgical theater, % providers that:**								
Primary provider A	0.0	1.2	15.2	11.0	47.3	.5	25.7	26.6
Secondary provider B	1.2	7.3	71.4	78.5	11.8	.5	74.3	73.4
Both perform and assist with VMMC operations depending on need	98.8	91.5	13.3	10.5	40.9	99.0	0.0	0.0
**Estimated number of VMMC procedures performed or assisted (career total):**								
Median	2430	1343	600	500	700	1500	360	400
(Interquartile range)	(500–4745)	(200–4185)	(200–2000)	(100–1000)	(275–1950)	(600–3000)	(70–1125)	(158–1000)
**Number of months performing VMMC for HIV prevention**								
Median	25 mo.	31 mo.	8 mo.	10 mo.	12 mo.	15 mo.	6 mo.	11 mo.
(Interquartile range)	(12–40)	(16–43)	(4–14)	(4–16)	(8–16)	(10–26)	(1–20)	(5–17)
**In the past 3 months % providers that performed VMMC:** Full-time C	64.7	45.1	80.0	78.5	1.1	50.0	33.8	13.8
**% of providers that received:**								
VMMC training in medical or nursing school	36.5	20.7	20.0	4.3	7.5	1.0	4.1	4.3
Additional formal training/continuing education (e.g., certificate training) in VMMC for HIV prevention	98.8	97.6	76.7	75.1	97.8	100	100	100
**Among those who had additional training:**	**n = 85**	**n = 82**	**n = 105**	**n = 160**	**n = 91**	**n = 206**	**n = 74**	**n = 94**
Mean number of days of additional training	21.2 days	20.8 days	5.7 days	5.7 days	13.9 days	11.5 days	6.8 days	7.0 days
(Standard deviation)	(20.5)	(13.9)	(3.5)	(2.8)	(5.2)	(1.6)	(1.5)	(0.2)

A Primary provider performs VMMC (removes foreskin).

B Secondary providers assist the primary surgical provider.

C Full-time defined as dedicated ≥90% of working hours to VMMC.

Table 3 describes the extent to which these four countries had adopted each of the six elements of VMMC efficiency. This issue is discussed in detail in the overview article [5] in this supplement. In summary, by 2012 countries had adopted 3–5 of the 6 efficiency elements. The forceps-guided surgical method and task-sharing were the only elements adopted by all countries (see Table 3). Data suggested that providers were aware of the nationally-recommended efficiency elements. For example, in 2012, 71% of providers in Kenya, 67% in South Africa, 88% in Tanzania, and 100% in Zimbabwe were aware that the forceps-guided method was the nationally-recommended surgical technique (data not shown).

**Table 3:** Summary of adoption of 6 efficiency elements across the four SYMMACS countries: 2011–2012.

	Kenya	South Africa	Tanzania	Zimbabwe
	2011–2012	2011–2012	2011–2012	2011–2012
Multiple bays in operating theatre		X/X	X/X	X/X
Purchase of pre-bundled kits with disposable instruments		X/X		X/X
Task-shifting	X/X		X/X	
Task-sharing	X/X	X/X	X/X	X/X
Surgical method: forceps-guided	X/X	X/X	X/X	X/X
Electrocautery to stop bleeding		X/X		(x)*/X

* Indicates partial adoption of efficiency element.

The results below indicate provider attitudes and perceptions regarding the six elements of surgical efficiency and other aspects of the VMMC scale-up.

### Task-shifting

Task-shifting (the use of trained clinical personnel that are not medical doctors to perform VMMC) is authorized and widely practiced in Kenya and Tanzania, whereas in South Africa and Zimbabwe only medical doctors are authorized to perform VMMC (see Table 1). Table 4 presents descriptive data on two provider attitudes toward task-shifting and four provider attitudes toward task-sharing; it tests for differences by cadre within each country/year and for differences by country.

**Table 4:** Provider attitudes toward task-shifting and task-sharing by cadre, country and year: descriptive statistics and crude odds ratios (2012 only).

Task-shifting: % of providers that strongly agree or agree with the following statements:				Task-sharing: % of providers that strongly agree or agree that is it acceptable for the secondary provider to:			
		Only Medical doctors perform VMMC	Primary provider present for the entire procedure	Prepare and scrub the patient	Administer local anesthesia	Dress the operating wound	Complete interrupted skin sutures
**Year**	N	%	%	%	%	%	%
**South Africa (reference)**							
2011							
Medical doctors	21	14.3	19.1	100.0	90.5	100.0	90.5
Non-medical doctors	84	27.4	26.2	97.7	96.5	98.8	92.8
2012							
Medical doctors	35	8.6	2.9*	100.0	94.3	100.0	94.3
Non-medical doctors	174	16.7	13.2	97.7	95.4	98.3	96.5
**Kenya**							
2011							
Medical doctors	0	—	—	—	—	—	—
Non-medical doctors	85	3.6	75.3	81.2	68.2	91.7	65.9
2012							
Medical doctors	0	—	—	—	—	—	—
Non-medical doctors	82	3.6	72.0	78.1	52.5	85.4	65.8
**Tanzania**							
2011							
Medical doctors	0	—	—	—	—	—	—
Non-medical doctors	93	9.7	91.4	97.9	97.7	100.0	99.9
2012							
Medical doctors	0	—	—	—	—	—	—
Non-medical doctors	202	2.5	75.3	97.5	97.5	100.0	99.6
**Zimbabwe**							
2011							
Medical doctors	19	15.8	5.3	100.0	84.2	100.0	78.9*
Non-medical doctors	55	3.6	1.8	100.0	94.5	100.0	94.6
2012							
Medical doctors	25	12.0	0.0	100.0	96.0	100.0	100.0
Non-medical doctors	69	5.8	1.5	100.0	95.7	100.0	97.1

* Fisher exact test p-value <0.10 (comparing cadre within country and year).

On the first attitude—“medical doctors are the only healthcare cadre who should be trained to perform adult VMMC”—relatively few providers in any country agreed with this statement and there was no statistical difference by cadre within each country and year. However, logistic regression results indicated differences by country (data not shown). Providers in Kenya and Tanzania were less likely than those in South Africa to agree with the statement, reflecting the fact that task-shifting is in widespread use in the countries less likely to agree. Zimbabwe did not differ from South Africa on this attitudinal variable.

In response to the open-ended questions, Zimbabwean and South African providers complained that part-time doctors created delays in program activity, as they often had other conflicting duties or would come late after frustrated clients had already left. The overwhelming recommendation by medical doctors and other clinical providers was that nurses should be trained to perform the entire procedure.

The second attitudinal variable, relating to rotation among multiple surgical bays, is worded to reflect an attitude favorable toward staying with a single patient from start to finish, and, by implication, negative toward rotation among multiple beds. The large majority of providers in Kenya and Tanzania agreed with the statement that “primary provider should be with the patient from the administration of anesthesia to the final dressing” (Table 4). However, results from the logistic regression (data not shown) indicated that both Kenya and Tanzania differed significantly from South Africa (the reference category) on this variable, whereas providers in Zimbabwe were even less likely than those in South Africa to agree with this attitude, reflecting a strong preference in Zimbabwe for the use of multiple beds.

### Task-sharing

Task-sharing refers to allowing providers other than medical doctors (described here as “secondary providers”) to conduct certain steps of the procedure. Where medical doctors must perform VMMC, nurses, clinical officers, and assistant medical officers (depending on country) can assist with tasks such as scrubbing the patient pre-op, administering the anesthesia, and completing suturing.

The four attitudinal questions are all worded such that agreement with the statement reflects approval of different steps in task-sharing. Across all countries and in both years, over three quarters of providers agreed with the statement that “it is acceptable for secondary providers to prepare and scrub the patient.” Similarly, the majority of providers in all countries in both years agreed that “it is acceptable for the secondary provider to administer local anesthesia.” High levels of agreement were observed in all countries and in both years on the statement that “it is acceptable for the secondary provider to dress the operating wound.” And in all countries except Kenya, over three quarters of providers in both years agreed that “it is acceptable for the secondary provider to complete the interrupted skin sutures.”

Logistic regression models were developed for testing for differences by country on the attitudinal variables related to task-sharing (data not shown). Regarding the attitude toward secondary providers administering local anesthesia, providers in Kenya were less likely to approve of this than their counterparts in South Africa. However, no other differences were detected by country or cadre. Similarly, providers in Kenya were less likely than those in South Africa to approve of having the secondary provider complete the interrupted skin sutures, in contrast to Tanzania where providers were even more open to task-sharing on this step.

### Surgical method

By the start of SYMMACS in 2011, all four countries had adopted forceps-guided as the surgical method of choice. Study findings indicated that the large majority of providers in all four countries preferred forceps-guided in both rounds of the survey (ranging from 73% to 95% in both years). However, in Kenya and Zimbabwe, we observed a decrease in 2012 (data not shown).

Despite the widespread preference for forceps-guided, many providers responded to the open-ended questions that they wanted training in other surgical methods for performing VMMC. They noted that this would avert the need to refer clients to city centers or hospitals in cases of penile conditions that require use of an alternative surgical method. Providers felt that such referrals would result in the loss of some potential VMMC clients from the system.

### Use of electrocautery instead of ligating sutures

In 2011 and 2012, the use of electrocautery to stop bleeding after removing the foreskin was in widespread use in South Africa and Zimbabwe, but was infrequent in Kenya and nonexistent in the Tanzanian program (thus, this set of questions was omitted in Tanzania). The vast majority of providers in South Africa, Kenya, and Zimbabwe (88–98%) believed that “electrocautery/diathermy is safe to use for homeostasis when performing adult male VMMC,” with no statistical difference between the countries (see Table 5). Furthermore, providers in all countries stated that electrocautery reduces the operating time significantly (with the percent agreeing ranging from 94% to 98% of providers in 2012), again with no statistically significant difference between countries for either year.

**Table 5:** Attitudes toward multiple surgical beds, electrocautery and pre-bundled kits, by country/and year.

	Kenya	South Africa	Tanzania	Zimbabwe
**All providers:**				
2011	82	105	93	74
2012	84	209	206	94
**Electrocautery (diathermy):**				
% of providers using electrocautery:				
2011	34.1	99.0	0.0	71.6**
2012	19.0	98.1	0.0	91.5**
**% of providers that report using electrocautery and strongly agree or agree with the following statements:**				
Electrocautery/diathermy is safe to use for hemostasis				
2012	87.5	97.0	—	97.7
Electrocautery decreases operating time significantly				
2011	92.9	89.5	—	98.1
2012	93.8	98.1	—	97.7
Electrocautery/diathermy compromises surgical sterility				
2011	32.2	27.9	—	3.8**
2012	18.8	38.0	—	0.0**
**Use of multiple surgical beds:**				
% of providers using multiple surgical beds				
2011	65.9	93.3	97.8	100.0**
2012	58.3	78.9	87.4	100.0**
% of providers prefer rotating between multiple surgical beds+				
2011	39.8	77.6	93.4	100.0**
2012	22.4	78.8	64.4	100.0**
**% of providers that strongly agree or agree that:**				
Using pre-bundled kits decreases the time needed to perform male circumcision:				
2011	98.8	93.3	100.0	98.6*
2012	97.6	96.7	72.8	100**
Using pre-bundled kits is an unnecessary expense:				
2011	1.2	10.5	30.2	13.5**
2012	3.6	10.0	12.7	8.5
I prefer assembling a surgical tray myself:				
2011	7.1	17.2	93.5	5.4**
2012	11.0	9.5	88.8	2.1**
If a clinic does use pre-bundled kits, the instruments should be reusable.				
2011	91.7	16.2	100.0	55.4**
2012	87.9	21.0	10.2	36.2**

+ As opposed to “attending to one patient at a time” or “no preference.”

* Chi square test p-value <0.05 (comparing countries within each year).

** Chi square test p-value <0.01 (comparing countries within each year).

Between 19% and 38% of providers in Kenya and South Africa (both years) believed that “electrocautery/diathermy compromises the sterility of the VMMC procedure,” compared to less than 4% of providers in Zimbabwe holding this view in 2011 and 0% in 2012. There was a statistically significant difference between countries, for both years, on this variable. In response to the open-ended questions, a number of providers in South Africa reported that diathermies did not work properly or that the equipment needed to perform the suturing was insufficient. In response to the open-ended questions, Kenyan providers strongly recommended training on the use of electrocautery.

### Rotation among multiple surgical bays

Rotation among multiple surgical beds refers to a practice whereby a team simultaneously utilizes two or more surgical beds to increase its efficiency and surgical outputs. As shown in the overview article [5], this practice was in widespread use in South Africa, Tanzania, and Zimbabwe, and the provider survey confirmed that the majority of providers (ranging from 64–100%, both years) preferred rotating among multiple surgical beds (Table 5). By contrast, in Kenya only 40% in 2011 and 22% in 2012 preferred multiple beds. There was a statistically significant difference between countries, for both years, on the reported preference for bed rotation. Kenyan providers cited a shortage of space, but the predominant reason seemed to be a sense of professional duty that the provider should stay with one patient from start to finish rather than pass him off to others.

### Bundling of supplies and tools

The practice of using purchased kits with pre-bundled supplies and disposable instruments is considered to increase surgical efficiency by giving the primary provider ready access to all the sterilized instruments and supplies needed at the start of the operation. Moreover, the use of disposable instruments averts the need for sterilizing instruments between operations in an autoclave. At the time of the survey, South Africa and Zimbabwe used purchased pre-bundled kits with disposable instruments; Kenya and Tanzania generally did not.

Table 5 presents data on provider attitudes toward use of pre-bundled kits with disposable instruments. The large majority of providers in all countries and in both years (73–100%) agreed that “using pre-bundled kits of instruments and supplies decreases the time needed to perform male circumcision,” and there was a statistically significant difference by country for both years. To the contrary, relatively few providers (1–30% in the different countries) felt that using pre-bundled kits of instruments and supplies is an unnecessary expense in VMMC clinics, with a significant difference between countries in 2011. The replies differed significantly for the remaining two attitudes related to kits. In Tanzania, 89–94% of providers reported that “I prefer assembling a surgical tray myself rather than using a pre-bundled VMMC kit,” compared to less than 18% in the other three countries in either year. Thus, a statistically significant difference was found between countries for both years. Tanzanian providers' answers to the open-ended questions indicated that they felt the latter practice ensured that they had everything needed for the procedure, as certain items were sometimes missing from pre-bundled kits.

Regarding approval of the use of reusable instruments, the countries varied greatly. On the extremes, approximately 90% of Kenyan providers in both years approved of reusable instruments, compared to approximately 20% in South Africa. In Tanzania, the percent favorable to reusable instruments dropped from 100% in 2011 to only 10% in 2012, mainly due to the fact that in that country, disposable kits were introduced in 2012 and providers found these extremely user-friendly. Again, a statistically significant difference in approval of reusable instruments was found between countries for both years.

## Discussion

This study adds to the scant literature on provider attitudes to VMMC. Research from other areas of public health has demonstrated that provider attitudes, preferences, and willingness to adhere to set guidelines – especially for new interventions – are critical for successful implementation of programs [8], [9], [10]. This study showed high concordance between each country's policies and provider attitudes toward the efficiency elements. Nonetheless, although providers generally endorse the six elements of surgical efficiency in VMMC programs, they may not necessarily agree with set guidelines. For example, many providers in Kenya and, to a lesser extent, in Tanzania did not endorse task-sharing given their belief that the provider has a professional obligation to remain with one client from the start to the end of the procedure. Where task-shifting is not authorized (South Africa and Zimbabwe), providers strongly embraced task-sharing.

Study findings suggest the need to consult providers and open a dialogue to ensure greater understanding of the rationale underlying certain policies to ensure effective implementation of VMMC efficiency elements. Involving service providers right at the formulation of guidelines is recognized as key to the successful adoption and implementation of interventions, especially where they are new [14]. Frustration over policies formulated in a “top-down” manner was not only expressed in this study, but has also been documented in others [6], [7], [11]. To maximize adherence, especially within a context where providers have their own preferences, there is a need to closely monitor the implementation of nationally-recommended policies [8], [9]. In this case, continuous training and especially refresher training will be crucial in ensuring adherence to VMMC efficiency elements. Of note, refresher courses were recommended by service providers.

SYMMACS findings have direct programmatic implications. Firstly, findings relating to preference of task-shifting by service providers (in combination with other factors) have led to a review of this VMMC element in Zimbabwe and South Africa. The endorsement of task-shifting in countries where it was not practiced presaged the change in policy current underway in Zimbabwe. As this article goes to press, the government of Zimbabwe is currently piloting task-shifting of VMMC to nurses.

Secondly, where the practices are not in place (Kenya and Tanzania), providers appear very open to receiving training in the use of electrocautery, as well as in other surgical methods (to use as a backup in case of medical contraindications for forceps-guided). Thirdly, providers in Kenya – which has the longest running program among the four countries – were much less likely to embrace the elements of task-sharing and bed rotation than their counterparts in the other three countries. For example, the strong sense of professional duty to complete the operation from start to finish rather than pass it off to others resulted in stark preference disparities between Kenyan providers and those from the other three countries. Thus, Kenyan providers felt that it was less acceptable for secondary providers to conduct crucial tasks such as administration of anesthesia and completing interrupted sutures. This philosophy also influenced attitudes around task-sharing even simpler tasks such as scrubbing and preparing the client. The philosophy reflects the approach of the Kenya program to have a far greater number of VMMC sites but less intense demand at each site; as a result, these providers may experience less pressure to adopt all elements of efficiency to be able to handle a larger volume of clients per day.

The strengths of this study include the following: it complements structured questions with responses to open-ended questions. Whereas quantitative data permitted comparisons between countries and over the two rounds of data collection, qualitative statements yielded a more nuanced understanding of provider perceptions and practices, highlighting the value of combining the two types of data [15], [16], [17]. Additionally, data were collected from four countries, which offered the opportunity to compare data from more than one setting. Furthermore, although the four countries have slightly different approaches to VMMC, we used standardized data collection tools, which ensured collection of uniform data for this multi-country study. Lastly, the same set of data was collected over two years, which offered us the opportunity to compare findings within and among countries over time.

A potential limitation of this study is social desirability bias—the tendency to provide responses thought to be more favorable or acceptable as opposed to being reflective of true thoughts or feelings [18]. Since the survey was administered at the clinics, albeit by interviewers not associated with the facility, it is possible that some providers found it difficult to articulate their real attitudes, especially if they felt that these contradicted national policy. Additionally, for various reasons we were unable to collect data from the same providers in both 2011 and 2012; this limited our ability to do analysis comparing outcomes by year.

In conclusion, national guidelines in each country have dictated the adoption (or not) of six elements of surgical efficiency in VMMC programs. The generally high degree of concordance between the policies of each country and provider attitudes towards these elements not only highlights the influence of the policy that is advocated and implemented in each country but also bodes well for compliance and effective implementation.
